# Effects of Exoskeleton Gait Training on Balance, Load Distribution, and Functional Status in Stroke: A Randomized Controlled Trial

**DOI:** 10.3389/fneur.2019.01344

**Published:** 2020-01-15

**Authors:** Anna Rojek, Anna Mika, Łukasz Oleksy, Artur Stolarczyk, Renata Kielnar

**Affiliations:** ^1^Physiotherapy Clinic RehaPlus, Kraków, Poland; ^2^Department of Clinical Rehabilitation, University of Physical Education in Krakow, Kraków, Poland; ^3^Physiotherapy and Sports Centre, Rzeszow University of Technology, Rzeszow, Poland; ^4^Oleksy Medical & Sports Sciences, Łańcut, Poland; ^5^Orthopaedic and Rehabilitation Department, Medical University of Warsaw, Warsaw, Poland; ^6^Medical College of Rzeszow University, Rzeszow, Poland

**Keywords:** ischemic stroke, exoskeleton, physiotherapy, balance, load distribution, functional status

## Abstract

**Background:** As a result of stroke, patients have problems with locomotion and transfers, which lead to frequent falls. Recovery after stroke is a major goal of rehabilitation, but it is difficult to choose which treatment method is most beneficial for stroke survivors. Recently, powered robotic exoskeletons are used in treatment to maximize the neural recovery of patients after stroke, but there are no studies evaluating the changes in balance among patients rehabilitated with an exoskeleton.

**Purpose:** The aim of this study was to evaluate the effects of Ekso GT exoskeleton-assisted gait training on balance, load distribution, and functional status of patients after ischemic stroke.

**Methods:** The outcomes are based on 44 patients aged 55–85 years after ischemic stroke who were previously randomly assigned into two groups: experimental (with Ekso GT rehabilitation) and control (with classical rehabilitation). At baseline and after 4 weeks of treatment, the patients were evaluated on balance, load distribution, and functional status using, respectively a stabilometric platform, the Barthel Index, and the Rivermead Mobility Index.

**Results:** In the experimental group, balance improved regarding the variables describing sway area as ellipse major and minor axes. In the control group, improvement was noted in sway velocity. After the therapy, total load distribution on feet in both groups showed a small and insignificant tendency toward reduction in the amount of uninvolved limb loading. In the control group, significant load transfer from the backfoot to the forefoot was noted. Both forms of rehabilitation caused significant changes in functional status.

**Conclusions:** Both training with the use of the Ekso GT exoskeleton and classical physiotherapy lead to functional improvement of patients after ischemic stroke. However, in the experimental group, improvement was observed in a larger number of categories, which may suggest potentially greater impact of treatment with the exoskeleton on functional status. Also, both forms of rehabilitation caused significant changes in balance, but we have noted some trends indicating that treatment with exoskeleton may be more beneficial for some patients. The load transfer from the backfoot to the forefoot observed in the control group was an unfavorable phenomenon. We suggest that the Ekso GT exoskeleton may be a promising tool in the rehabilitation of patients after stroke.

**Trial registration:** Trial ID ACTRN12616000148471

## Introduction

Stroke is the third leading cause of death worldwide and is the most common cause of disability among adults (1, 2). As a result of stroke, patients have problems with locomotion and transfers, which lead to frequent falls. People with hemiparesis have uneven distribution of body mass between the sides of the body, causing balance and coordination disorders, deep and superficial sensation, increased muscle tone, and fear of falling (2, 3). Patients have problems with lack of normal postural muscle tone, and proper reciprocal innervation as well as normal, automatic movement patterns and balance reactions (4). Some studies have reported that balance alterations significantly limit the physical activity of stroke patients, which may be the reason for deconditioning of patients in the chronic phase and reduction in their gait possibilities as well as other activities of daily living (5). That is why gait rehabilitation and also balance therapy are very important in improving the quality of everyday and social life of those patients (6).

Gait training may improve not only strength, endurance, and coordination of the lower limbs but also the entire body of the patient, influencing general fitness and endurance, balance, normalization of muscle tone, and functional improvement (7). The Barthel Index (BI) and Rivermead Mobility Index (RMI) tests are considered to be proper criteria for assessing a patient's functional state after stroke and good indicators of the effectiveness of the applied therapy (8, 9).

Recovery after stroke is a major goal of rehabilitation, but it is difficult to choose which treatment method is most beneficial for stroke survivors. Recently, powered robotic exoskeletons are used in treatment to maximize the neural recovery of patients after stroke (10, 11). However, in a review paper, Louie and Eng (12) have reported that only four different types of powered exoskeletons have been studied among a small number of stroke patients, and the published data were controversial. Moreover, in the available literature, there are no studies evaluating the changes in balance among patients rehabilitated with an exoskeleton. Most authors have reported various aspects of walking, and only a few papers have presented data concerning changes in balance. Additionally, most of the studies used subjective tools such as the Berg Balance Scale (13, 14). There is a lack of studies in which changes in balance and load distribution due to rehabilitation with the exoskeleton would be examined using an objective tool—stabilometric platform; therefore, this study undertakes this task for the first time.

The aim of this study was to evaluate the effectiveness of rehabilitation with Ekso GT exoskeleton in patients after ischemic stroke and to compare this type of therapy with the classical model of rehabilitation. The novelty of this study was the verification of the robot-assisted gait training effects on balance, load distribution, and functional status of stroke patients.

## Materials and Methods

### Participants

**Figure 1:**
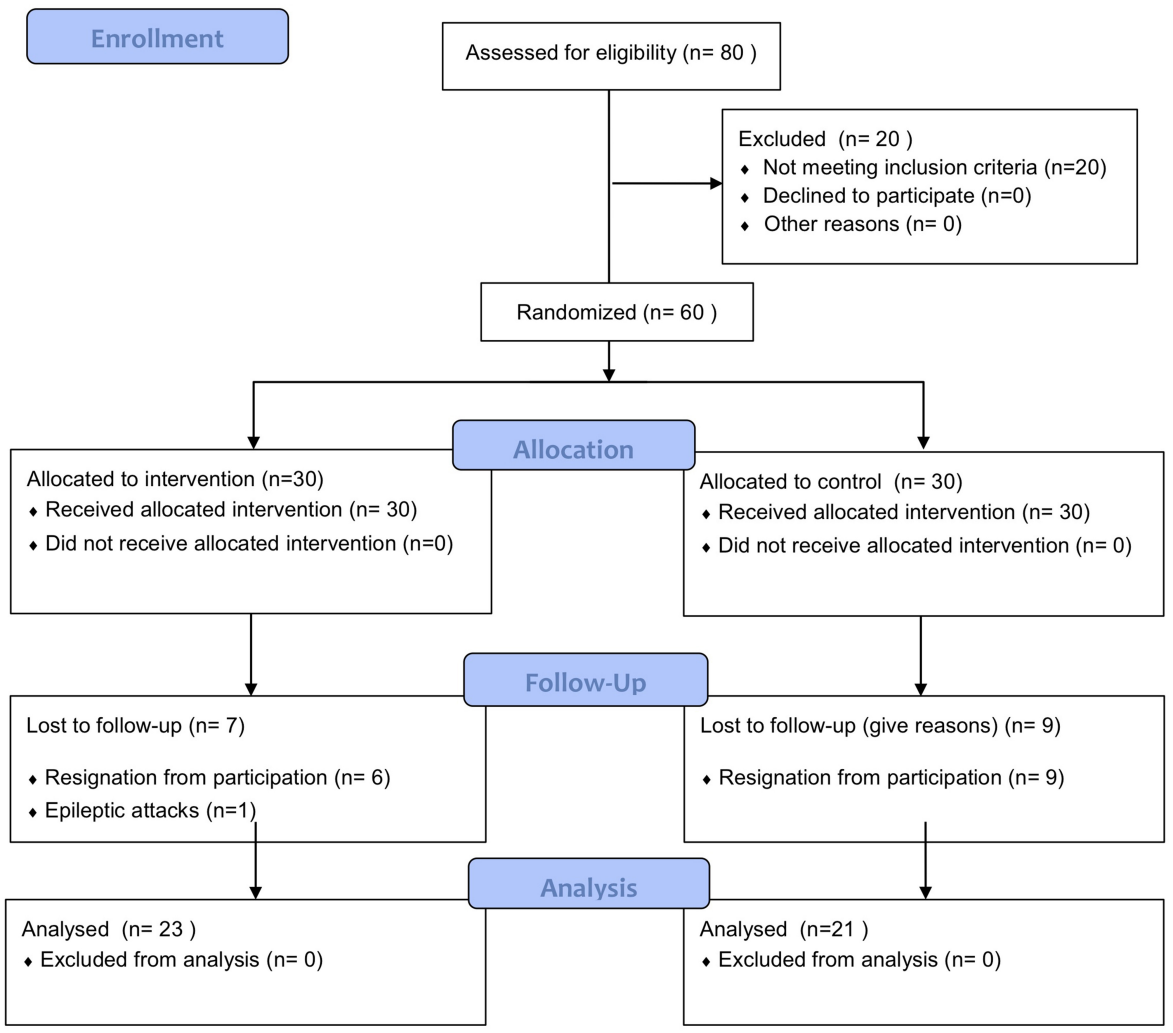
CONSORT flow diagram.

This randomized controlled trial included a group of 44 participants (19 women and 25 men) aged 55–85 years (69 ± 7) after the ischemic stroke incident which took place not earlier than 1 year before enrolment in the study (Figure 1). Patients were recruited from three hospitals with a neurological rehabilitation department or subunit.

Patient characteristics are presented in Table 1. Patients were randomly divided into two groups:

- Group 1 (experimental): *n* = 23, including patients who underwent rehabilitation with the Ekso GT exoskeleton
- Group 2 (control): *n* = 21, including patients who underwent classical rehabilitation after stroke.

**Table 1:** Patient characteristics.

	**Experimental group**	**Control group**
Number of participants (*n*)	23	21
Sex	10 women, 13 men	9 women, 12 men
Age (years)	55–85 (69 ± 8)	59–82 (70 ± 6)
Stroke type	Ischemic	Ischemic
Left-sided paresis (*n*)	12	12
Right-sided paresis (*n*)	11	9
Time since stroke (months)	4–12	5–12

The exclusion criteria are as follows:

- Second or subsequent stroke incident
- Disproportion in the length of lower limbs >2 cm
- Fixed bony and joint contractures as well as articular deformations
- Inflammatory changes on the skin and open skin lesions in the area of the trunk or lower limbs
- Spasticity of muscles of the lower limbs >3 according to the Modified Ashworth Scale
- Aphasia making communication with the patient impossible
- Severe amblyopia or hearing loss
- Limitation in lower limbs' range of motion which restricts full upright standing position in exoskeleton or not allowing him/her to move from a standing to sitting position or vice versa
- Lack of sufficient strength in the upper limbs, limiting the patient's ability to maintain balance while walking with a walker or crutches
- Reduced standing tolerance due to orthostatic hypotension
- Severe osteoporosis preventing the patient from assuming a safe standing position or potentially increasing the risk of fracture when standing or walking.

The qualification procedure included the following:

- Medical qualification
  Patients from both groups were subjected to medical qualification conducted by a specialist physician who stated that there were no contraindications for physical rehabilitation.
- Qualification of patients to train with the Ekso GT exoskeleton
  The physiotherapist, who operated the exoskeleton, was responsible for this qualification.

It included the following measurements:

- Range of motion in the shoulder, elbow, wrist, hip, knee, and ankle joints
- Muscle spasticity responsible for movements in the shoulder, elbow, wrist, hip, knee, and ankle joints using the Modified Ashworth Scale
- Skin condition (control of the presence of abrasions, wounds, etc.)
- Linear measurements of hip width and thigh and lower leg length
- Collection of history regarding the patient's state of health, including other medical problems such as the presence of orthostatic hypotension or osteoporosis.

The patients were informed in detail about the research protocol and gave their written informed consent to participate in the study. If patient was unable to sign it personally, a family member did this on his/her behalf. The approval of the Ethical Committee of Regional Medical Chamber in Krakow was obtained before the study.

Before starting the intervention, patients were randomly allocated to the experimental or control group by an independent researcher using the sealed envelopes method.

### Experimental Procedures

All measurements were performed twice, at baseline and after 4 weeks of treatment by a blinded investigator.

### Evaluation of Balance and Load Distribution

Measurements were taken using the zebris FDM-S baroresistive platform (zebris, Germany), which allowed us to examine balance and load distribution between the right and left feet for the forefoot and backfoot, respectively. The platform used in this study was baroresistive in construction, because it has baroresistive sensors. But it was stabilometric in function because it measures balance and load distribution in static and dynamic activities. Calibration of the platform was conducted prior to data collection based on the manufacturer's instructions. During the measurement, the patient stands barefoot, upright in the middle of the platform, with arms to his/her sides in a relaxed, habitual position, remaining motionless for 30 s and looking straight ahead. The measurement was repeated twice: the first time with open eyes and the second with eyes closed. Between measurements, the patient rested in a sitting position for as long as she/he needed. During the examination, patients did not see a computer monitor displaying the right- and left-foot load values.

The evaluated variables are as follows:

The ellipse was defined as 95% of the confidence area around the center-of-pressure (COP) sway area of the whole body (mm^2^).

- COP path length (mm)—sway path length
- COP average velocity (mm/s)
- Length of minor axis (mm)—ellipse width
- Length of major axis (mm)—ellipse height
- Angle between *Y* and major axes (degrees)—ellipse angle: indicates orientation of the direction of the longitudinal axis regarding the ellipse compared to the longitudinal axis of the platform
- COP deviation *X* (mm)—COP horizontal standard deviation
- COP deviation *Y* (mm)—COP vertical standard deviation.

Load distribution under the feet was measured as the force of Newton per square centimeter acting on the plate sensors. Load distribution between the left and right feet was expressed in percentages.

- Total load (%)—the average percentage pressure distribution of the left and right feet
- Forefoot load (%)—the average percentage pressure distribution of the left and right forefeet
- Backfoot load (%)—the average percentage pressure distribution of the left and right backfeet.

### Functional Status Evaluation

#### Rivermead Mobility Index

The RMI determines the degree of disability and mobility of patients after stroke, testing functional abilities such as gait, balance, or transfers. An end result of 0 means that the examined person is not able to perform any of the tested activities correctly and shows a high level of disability (15, 16). Previously reported inter-rater and intra-rater reliability of RMI was excellent [intraclass correlation coefficients (ICCs) 0.97 and 0.99] (17, 18).

#### Barthel Index

The BI was used to determine the functional status of the examined patients based on the specific daily activities. The scale is a reflection of what the patient is able to do, while the purpose of its use is to determine the degree of the patient's independence. In this test, patients can obtain a maximum of 100 points (19, 20). Previously reported ICC representing inter-rater reliability for the BI was 0.99. For intra-rater reliability, the value of the ICC was 0.99 (21).

In both questionnaires, patients answered the questions themselves as much as possible. In the case of speech or cognitive disorders, the responses were provided by relatives, caregivers, or therapists working with the patient.

### Walking Time and Number of Steps Monitored With the Ekso GT Exoskeleton

In the experimental group, the functions monitored by the Ekso GT exoskeleton during therapy were measured at baseline and after each week of treatment: walking time and the number of steps performed during gait in the exoskeleton.

### Therapeutic Interventions

In both groups, rehabilitation was conducted five times a week, for 4 weeks. Each patient was treated daily by the same licensed physiotherapist. Therapy dose was equal between the two groups.

#### Experimental Group

Patients were trained using the Ekso GT exoskeleton. The duration of a single therapeutic session was 45 min. In addition to gait training with the Ekso GT exoskeleton, patients from this group received occupational therapy and individually selected physical therapy for 60 min/day.

For training, the examined person was brought with assistance or, if necessary, in a wheelchair. First of all, the patient was moved to a chair (chair height: 47 cm), on which there was a previously fitted exoskeleton—with a properly adjusted thigh and lower leg length for the patient. The device was fastened to the patient's body, and therapy was started. The patient was positioned vertically by the device; then gait training was begun. This took place on a flat surface—along the hospital corridor. In case of fatigue, the patient had the opportunity to rest on a chair. The first therapy session consisted of body mass shifting between sides. The training was terminated when the patient's fatigue was too great or when 45 min had elapsed.

Parameters of gait were monitored and, if necessary, modified and adapted to the needs as well as individual capabilities of patients. The exoskeleton function “Variable Assist” was used as often as possible. It is an adaptive program supporting both sides of the body, where the right and left sides can be controlled independently, allowing independent support of each limb in a patient after stroke. The amount of support can be automatically or manually adjusted^1^.

#### Control Group

Patients from the control group were treated with classical rehabilitation, which included individual exercises with a therapist, verticalization and gait (45 min); group exercises improving general fitness; occupational therapy; and individually selected elements of physical therapy (60 min).

The patient was brought to the gym, where she/he worked with the physical therapist individually. The patient did the exercises on a therapeutic table to strengthen his/her trunk and lower limbs and then moved to higher positions such as sitting on the table or standing, depending on his/her current possibilities. She/he also did balance exercises and was taught to walk on a flat surface, with the therapist's assistance. If the patient's condition allowed for it, after completing the exercises, she/he went to train on a stationary bicycle, where resistance was adjusted to the patient's own abilities. Then, the patient went on to physical therapy (mainly electro-stimulation and whirlpool bath). After these procedures, the patient participated in occupational therapy classes.

### Statistical Analysis

Statistical analysis was performed using the STATISTICA 12.0.PL software. The normality of the distribution of variables in groups was checked using the Shapiro–Wilk test. The significance of changes in balance and load distribution was determined using the two-way ANOVA (ANOVA group × time). The changes in functional status evaluation variables (BI and RMI) were assessed with the non-parametric chi-square test. The Bonferroni correction was used for multiple comparisons. The effect size was calculated using Cohen's *d*, analyzed and discussed in accordance with the previous studies (22–24). The differences were considered statistically significant if the level of test probability was lower than the assumed level of significance (*p* < 0.05). The paired *t*-test power analysis of exercise influence determined that at least 20 subjects were required to obtain a power of 0.8 at the two-sided level of 0.05 with the effect size of *d* = 0.6. This analysis was based on data derived from previous literature (25, 26).

## Results

### Balance and Load Distribution

#### Balance With Eyes Open

The COP path length in both groups did not change significantly after therapy (*p* > 0.05). However, in the control group, there was a noticeable trend toward increasing the length of the path with strong effect size (ES = 0.7). A significant difference was observed after therapy between the study groups. The average COP velocity did not change significantly in any of the groups (*p* > 0.05), but there was a significant difference between groups after treatment with a tendency toward an increase in the control group (ES = 0.74). After therapy in the experimental group, a non-significant tendency showing improvement in the ellipse smaller axis (ES = 0.54) and ellipse larger axis (ES = 0.41) was noted, while in the control, a non-significant trend toward deterioration of these parameters was observed (ES = 0.89; ES = 0.76). The COP deviation *X* (in the frontal plane) in the experimental group improved significantly, while in the control group, the trend was in the opposite direction (ES = 0.34). The COP deviation *Y* (in the sagittal plane) in the experimental group improved significantly, while in the control group, it remained unchanged (*p* > 0.05) (Table 2).

**Table 2:** Comparison of balance and load distribution with eyes open at baseline and after therapy.

**Outcome measure**		**Experimental group**	***p*^**a**^**	**ES^**a**^**	**Control group**	***p*^**a**^**	**ES^**a**^**	***p*^**b**^**	**ES^**b**^**
COP path length (mm)	Baseline	712 ± 540	n.s.	0.17	726 ± 428	n.s.	0.70	n.s.	
	Post	607 ± 657			1,114 ± 565			**0.04**	**0.82**
COP average velocity (mm/s)	Baseline	23 ± 18	n.s.	0.15	25 ± 14	n.s.	0.74	n.s.	
	Post	20 ± 21			37 ± 18			**0.03**	**0.36**
Length of minor axis (mm)	Baseline	34 ± 24	n.s.	0.54	23 ± 9	n.s.	0.89	n.s.	
	Post	23 ± 15			33 ± 13			n.s.	**0.71**
Length of major axis (mm)	Baseline	67 ± 43	n.s.	0.41	38 ± 12	n.s.	0.76	n.s.	
	Post	52 ± 28			52 ± 23			n.s.	0.01
Angle to major axis (degree)	Baseline	41 ± 27	n.s.	0.31	45 ± 23	n.s.	0.57	n.s.	
	Post	33 ± 23			32 ± 22			n.s.	0.04
Deviation *X* (mm)	Baseline	50 ± 34	**0.04**	**0.54**	30 ± 21	n.s.	0.34	n.s.	
	Post	33 ± 28			39 ± 30			n.s.	0.20
Deviation *Y* (mm)	Baseline	48 ± 27	**0.04**	**0.51**	20 ± 9	n.s.	0.29	**0.004**	
	Post	35 ± 23			16 ± 17			**0.03**	**0.93**
Forefoot load involved (%)	Baseline	58 ± 28	n.s.	0.07	53 ± 21	n.s.	0.46	n.s.	
	Post	60 ± 26			63 ± 22			n.s.	0.12
Forefoot load uninvolved (%)	Baseline	27 ± 21	n.s.	0.10	39 ± 10	n.s.	0.44	n.s.	
	Post	29 ± 16			43 ± 8			**0.01**	**1.10**
Backfoot load involved (%)	Baseline	41 ± 28	n.s.	0.07	46 ± 21	n.s.	0.41	n.s.	
	Post	39 ± 26			37 ± 22			n.s.	0.08
Backfoot load uninvolved (%)	Baseline	72 ± 21	n.s.	0.10	60 ± 10	n.s.	0.33	n.s.	
	Post	70 ± 16			57 ± 8			**0.01**	**1.02**
Total load involved (%)	Baseline	33 ± 19	n.s.	0.11	36 ± 10	n.s.	0.32	n.s.	
	Post	35 ± 15			40 ± 14			n.s.	0.34
Total load uninvolved (%)	Baseline	66 ± 19	n.s.	0.11	63 ± 10	n.s.	0.32	n.s.	
	Post	64 ± 15			59 ± 14			n.s.	0.34

*p^a^–p-value between baseline and post-therapy within each group*.

*p^b^–p-value between study groups*.

*ES^a^–effect size (Cohen d) within each group*.

*ES^b^–effect size (Cohen d) between study groups*.

*COP—center of pressure*.

*n.s.—non-significant*.

*Values are expressed as mean ± SD*.

*Statistically significant results are marked in bold*.

#### Balance With Closed Eyes

The COP path length and COP velocity did not change significantly after therapy in the groups (*p* > 0.05). The length of the minor and major ellipse axes in both groups did not change significantly (*p* > 0.05). But, in the experimental group, a tendency to improve was observed (ES = 0.65). Also, after therapy, the difference between groups was non-significant, but with effect size (ES = 0.60). COP deviation *X* (in the frontal plane) in both groups did not change significantly (*p* > 0.05). COP deviation *Y* (in the sagittal plane) showed lower values after therapy, but the change was only significant in the control group (ES = 0.86). There was also a significant difference between groups after therapy (ES = 1.09) (Table 3).

**Table 3:** Comparison of balance and load distribution with eyes closed at baseline and after therapy.

**Outcome measure**		**Experimental Group**	***p*^**a**^**	**ES^**a**^**	**Control Group**	***p*^**a**^**	**ES^**a**^**	***p*^**b**^**	**ES^**b**^**
COP path length (mm)	Baseline	847 ± 554	n.s.	0.04	774 ± 491	n.s.	0.38	n.s.	
	Post	837 ± 619			938 ± 359			n.s.	0.19
COP average velocity (mm/s)	Baseline	28 ± 16	n.s.	0.05	26 ± 16	n.s.	0.35	n.s.	
	Post	29 ± 19			31 ± 12			n.s.	0.12
Length of minor axis (mm)	Baseline	32 ± 17	n.s.	0.37	24 ± 19	n.s.	0.12	n.s.	
	Post	26 ± 15			22 ± 13			n.s.	0.28
Length of major axis (mm)	Baseline	68 ± 44	n.s.	0.65	47 ± 45	n.s.	0.32	n.s.	
	Post	46 ± 18			36 ± 15			n.s.	0.60
Angle to major axis (degree)	Baseline	53 ± 26	n.s.	0.33	45 ± 28	n.s.	0.43	n.s.	
	Post	44 ± 28			34 ± 22			n.s.	0.39
Deviation *X* (mm)	Baseline	38 ± 32	n.s.	0.09	31 ± 23	n.s.	0.17	n.s.	
	Post	35 ± 33			36 ± 34			n.s.	0.02
Deviation *Y* (mm)	Baseline	46 ± 31	n.s.	0.27	25 ± 10	0**.01**	0**.86**	n.s.	
	Post	38 ± 27			14 ± 15			**0.03**	**1.09**
Forefoot load involved (%)	Baseline	53 ± 29	n.s.	0.08	53 ± 21	n.s.	0.46	n.s.	
	Post	51 ± 19			63 ± 22			n.s.	0.58
Forefoot load uninvolved (%)	Baseline	28 ± 23	n.s.	0.04	35 ± 10	n.s.	0.52	n.s.	
	Post	27 ± 18			40 ± 9			**0.04**	**0.91**
Backfoot load involved (%)	Baseline	46 ± 29	n.s.	0.08	46 ± 21	n.s.	0.46	n.s.	
	Post	48 ± 19			36 ± 22			n.s.	0.58
Backfoot load uninvolved (%)	Baseline	71 ± 23	n.s.	0.04	64 ± 10	n.s.	0.52	n.s.	
	Post	72 ± 18			59 ± 9			**0.04**	**0.91**
Total load involved (%)	Baseline	36 ± 21	n.s.	0.09	33 ± 14	n.s.	0.42	n.s.	
	Post	38 ± 19			39 ± 14			n.s.	0.05
Total load uninvolved (%)	Baseline	63 ± 21	n.s.	0.09	66 ± 14	n.s.	0.42	n.s.	
	Post	61 ± 19			60 ± 14			n.s.	0.05

*p^a^–p-value between baseline and post-therapy within each group*.

*p^b^–p-value between study groups*.

*ES^a^–effect size (Cohen d) within each group*.

*ES^b^–effect size (Cohen d) between study groups*.

*COP—center of pressure*.

*n.s.—non-significant*.

*Values are expressed as mean ± SD*.

*Statistically significant results are marked in bold*.

#### Load Distribution With Open Eyes

Total load distribution in both groups did not change significantly after therapy (*p* > 0.05). After therapy, the forefoot load in both groups did not change significantly (*p* > 0.05), but in the control group, there was a non-significant tendency to increase forefoot loading of both the involved (ES = 0.46) and uninvolved (ES = 0.44) sides. There was also a significant difference between groups after therapy on the uninvolved side (ES = 1.10). After therapy, in the control group, backfoot load showed a non-significant tendency to reduce the load on the involved (ES = 0.41) and uninvolved (ES = 0.33) sides. There was also a significant difference between groups after therapy (ES = 1.02) (Table 2).

#### Load Distribution With Closed Eyes

There were no significant differences in the experimental and control groups between baseline and post-therapy total load values (*p* > 0.05) or between groups after 4 weeks of treatment (*p* > 0.05). Forefoot load increased after therapy on both the involved (ES = 0.41) and uninvolved (ES = 0.52) sides. In the experimental group, there were no significant changes after treatment (*p* > 0.05). Between groups, differences were non-significant after therapy, but with strong effect size regarding the uninvolved (ES = 0.91) and involved (ES = 0.58) sides. The increased load of the forefoot appeared simultaneously along with a decreased backfoot load. A non-significant trend was noted in the control group with ES = 0.46 for the involved side and ES = 0.52 for the uninvolved side. After therapy, differences between groups were significant for the uninvolved side (ES = 0.91) and non-significant for the involved side (ES = 0.58) (Table 3).

### Functional Status Evaluation

#### Barthel Index

Despite the fact that this study was randomized, the results showed some significant differences between groups at baseline in favor of the control group. After therapy, significant improvement was observed in all categories in the experimental group, while in the control group, significant changes were noted only for three categories of BI. Furthermore, significant differences between groups were observed at baseline in some categories and after therapy, but overall, the improvement in functional status was stronger in the experimental group (Table 4).

**Table 4:** Comparison of the Barthel Index at baseline and after therapy.

**Outcome measure**		**Experimental group**	**χ^**2a**^**	***p*^**a**^**	**Control group**	**χ^**2a**^**	***p*^**a**^**	**χ^**2b**^**	***p*^**b**^**
Feeding	Baseline	5 ± 5 (0–5)	**10**	**0.001**	5 ± 5 (5–10)	2	n.s.	**4.9**	**0.02**
	Post	5 ± 5 (5–10)			10 ± 5 (5–10)			0.34	n.s.
Bathing	Baseline	0 ± 0 (0–0)	**5**	**0.02**	0 ± 5 (0–5)	2	n.s.	3.2	n.s.
	Post	0 ± 5 (0–5)			5 ± 5 (0–5)			1.42	n.s.
Grooming	Baseline	0 ± 0 (0–0)	**12**	**0.0005**	5 ± 0 (5–5)	1	n.s.	**17.8**	**0.0001**
	Post	5 ± 5 (0–5)			5 ± 0 (5–5)			0.26	n.s.
Dressing	Baseline	0 ± 5 (0–5)	**11**	**0.0009**	5 ± 5 (5–10)	3	n.s.	**12.3**	**0.004**
	Post	5 ± 5 (0–5)			5 ± 5 (5–10)			**6.3**	**0.01**
Bowel control	Baseline	5 ± 5 (0–5)	**10**	**0.001**	10 ± 0 (10–10)	1	n.s.	0	n.s.
	Post	10 ± 5 (5–10)			10 ± 0 (10–10)			0	n.s.
Bladder control	Baseline	5 ± 5 (0–5)	**10**	**0.001**	10 ± 0 (10–10)	1	n.s.	0	n.s.
	Post	10 ± 5 (5–10)			10 ± 0 (10–10)			0	n.s.
Toilet use	Baseline	5 ± 5 (0–5)	**8**	**0.004**	10 ± 5 (5–10)	**5**	**0.02**	**11.8**	**0.0006**
	Post	5 ± 5 (5–10)			10 ± 5 (5–10)			**7.37**	**0.006**
Transfers (bed to chair and back)	Baseline	5 ± 0 (5–5)	**11**	**0.0009**	15 ± 10 (5–15)	**7**	**0.008**	**7.3**	**0.006**
	Post	10 ± 10 (5–15)			15 ± 5 (10–15)			**4.3**	**0.03**
Mobility (on level surface)	Baseline	0 ± 0 (0–0)	**7**	**0.008**	15 ± 15 (0–15)	3	n.s.	**4.45**	**0.034**
	Post	0 ± 10 (0–10)			15 ± 15 (0–15)			3.19	n.s.
Stairs	Baseline	0 ± 0 (0–0)	**7**	**0.008**	5 ± 10 (0–10)	1	n.s.	**4.45**	**0.03**
	Post	0 ± 5 (0–5)			5 ± 10 (0–10)			2.27	n.s.
Total	Baseline	25 ± 25 (15–40)	**21**	**0.00001**	85 ± 25 (15–50)	**7**	**0.006**	**13**	**0.0003**
	Post	50 ± 35 (25–70)			85 ± 50 (50–100)			**5.7**	**0.01**

*p^a^–p-value between baseline and post-therapy within each group*.

*p^b^–p-value between study groups*.

*χ^2a^–chi-square test value within each group*.

*χ^2b^–chi-square test value between study groups*.

*n.s.—non-significant*.

*Values are expressed as median ± quantile range (lower quantile–upper quantile)*.

*Statistically significant results are marked in bold*.

#### Rivermead Mobility Index

Similarly, as was observed in the case of the BI, the results of the RMI showed some significant differences between groups at baseline in favor of the control group. After therapy, significant improvement was observed in the experimental group for most of the categories, while in the control group, significant changes were noted only for five categories of RMI. Also, significant differences between groups were observed after therapy, but overall, the improvement in functional status was stronger in the experimental group (Table 5).

**Table 5:** Comparison of the Rivermead Mobility Index at baseline and after therapy.

**Outcome measure**		**Experimental group**	**χ^**2a**^**	***p*^**a**^**	**Control group**	**χ^**2a**^**	***p*^**a**^**	**χ^**2b**^**	***p*^**b**^**
RMI 1	Baseline	1 ± 0(1−1)	2.6	n.s.	1 ± 0(1−1)	1	n.s.	0	n.s.
	Post	1 ± 0(1−1)			1 ± 0(1−1)			0	n.s.
RMI 2	Baseline	0 ± 1(0−1)	**12**	**0.0005**	1 ± 1(0−1)	**6**	**0.01**	**7.3**	**0.006**
	Post	1 ± 0(1−1)			1 ± 0(1−1)			0	n.s.
RMI 3	Baseline	1 ± 0(1−1)	2	n.s.	1 ± 0(1−1)	2	n.s.	0	n.s.
	Post	1 ± 0(1−1)			1 ± 0(1−1)			0	n.s.
RMI 4	Baseline	0 ± 0(0−0)	**8**	**0.004**	1 ± 1(0−1)	**5**	**0.02**	**7**	**0.006**
	Post	1 ± 1(0−1)			1 ± 0(1−1)			0	n.s.
RMI 5	Baseline	0 ± 0(0−0)	0.6	n.s.	1 ± 1(0−1)	**4**	**0.04**	**5.8**	**0.01**
	Post	0 ± 1(0−1)			1 ± 0(1−1)			0	n.s.
RMI 6	Baseline	0 ± 1(0−1)	**8**	**0.004**	1 ± 1(0−1)	**4**	**0.04**	4.3	0.03
	Post	1 ± 1(0−1)			1 ± 0(1−1)			0	n.s.
RMI 7	Baseline	0 ± 0(0−0)	**4.5**	**0.03**	1 ± 1(0−1)	3	n.s.	**11.8**	**0.0006**
	Post	0 ± 1(0−1)			1 ± 1(0−1)			0	n.s.
RMI 8	Baseline	0 ± 0(0−0)	**4.5**	**0.03**	1 ± 1(0−1)	0	n.s.	**7.4**	**0.006**
	Post	0 ± 1(0−1)			1 ± 1(0−1)			0.8	n.s.
RMI 9	Baseline	0 ± 0(0−0)	**4**	**0.04**	0 ± 1(0−1)	3	n.s.	3.4	n.s.
	Post	0 ± 1(0−1)			1 ± 1(0−1)			2.2	n.s.
RMI 10	Baseline	0 ± 0(0−0)	1.8	n.s.	0 ± 1(0−1)	2	n.s.	**10.9**	**0.0009**
	Post	0 ± 0(0−0)			1 ± 1(0−1)			**7.4**	**0.006**
RMI 11	Baseline	0 ± 0(0−0)	**8**	**0.004**	1 ± 1(0−1)	1	n.s.	**12.7**	**0.0004**
	Post	0 ± 1(0−1)			1 ± 1(0−1)			1.4	n.s.
RMI 12	Baseline	0 ± 0(0−0)	3	n.s.	1 ± 1(0−1)	0	n.s.	**16**	**0.0001**
	Post	0 ± 0(0−0)			1 ± 1(0−1)			**7.8**	**0.005**
RMI 13	Baseline	0 ± 0(0−0)	3	n.s.	0 ± 1(0−1)	**5**	**0.02**	2.2	n.s.
	Post	0 ± 1(0−1)			1 ± 1(0−1)			**4.4**	**0.03**
RMI 14	Baseline	0 ± 0(0−0)	3	n.s.	0 ± 1(0−1)	0	n.s.	**4.9**	**0.02**
	Post	0 ± 1(0−1)			0 ± 1(0−1)			2.1	n.s.
RMI 15	Baseline	0 ± 0(0−0)	1	n.s.	0 ± 0(0−0)	0	n.s.	0	n.s.
	Post	0 ± 0(0−0)			0 ± 0(0−0)			0	n.s.
RMI total	Baseline	3 ± 5(1−6)	**17**	**0.0003**	10 ± 12(2−14)	0.6	n.s.	3.2	n.s.
	Post	6 ± 7(3−10)			13 ± 8(6−14)			1.4	n.s.

*p^a^–p-value between baseline and post-therapy within each group*.

*p^b^–p-value between study groups*.

*χ^2a^–chi-square test value within each group*.

*χ^2b^–chi-square test value between study groups*.

*n.s.—non-significant*.

*Values are expressed as median ± quantile range (lower quantile–upper quantile)*.

*Statistically significant results are marked in bold*.

*RMI 1—turning from the back to the side without help*.

*RMI 2—independent transition from lying in bed to sitting on the edge of the bed*.

*RMI 3—sitting on the edge of the bed alone, without holding on to anything for 10 s*.

*RMI 4—getting up from the chair in <15 s (using a hand or help if necessary) and the ability to maintain a standing position for the next 15 s*.

RMI 5—observation of the patient standing alone, without help for 10 s.

*RMI 6—moving from bed to chair and back without any help*.

*RMI 7—10-m walking distance, with an orthopedic aid if necessary, but without anyone's help*.

*RMI 8—independently climbing up a stair (step), without help*.

*RMI 9—walking outside, on pavement*.

*RMI 10—independent walking inside, without a splint, stabilizer, or anyone's help*.

*RMI 11—walking 5 m to lift something that fell to the floor and return*.

*RMI 12—walking on uneven ground (grass, gravel, earth, snow, and ice) without help*.

*RMI 13—independently getting into the shower or into the bathtub and bathing without supervision*.

*RMI 14—going up and down four stairs without handrails, but using an aid if necessary*.

*RMI 15—running 10 m in 4 s without limping; fast gait is acceptable*.

*RMI total—total score*.

### Walking Time and Number of Steps Monitored With the Ekso GT Exoskeleton

**Figure 2:**
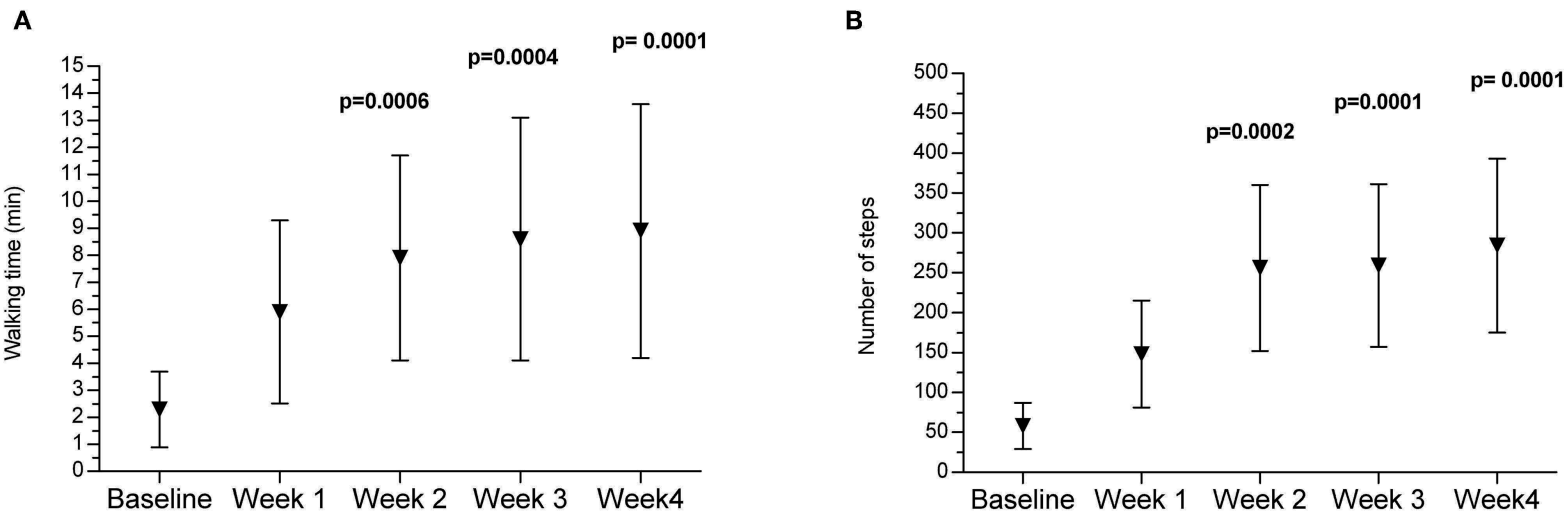
Changes in walking time **(A)** and number of steps **(B)** monitored with the Ekso GT during the therapy in the experimental group. *p*—*p*-value between baseline and 2, 3, and 4 week of therapy.

In the experimental group, during the 2 weeks of treatment with the Ekso GT exoskeleton, walking time increased significantly, and the following significant increase was observed after the third and fourth weeks of training (Figure 2A). Similar changes were noted in the number of steps performed by patients during a training session. A significant increase was observed after 2 weeks with a subsequent increase after the third and fourth weeks (Figure 2B).

## Discussion

The most important observations from this study were that both training with the Ekso GT exoskeleton and the use of classical physiotherapy led to functional improvement of patients after ischemic stroke. Both forms of rehabilitation caused significant changes in balance and functional status. However, none of the methods used was clearly better than the other. We have noted some trends showing that treatment with the exoskeleton may be more beneficial in some patients. In the experimental group, balance measured with open eyes improved, especially regarding variables describing sway area as ellipse major and minor axes. But in those patients, after 4 weeks of treatment with the exoskeleton, we did not observe an increase in sway velocity, which may indicate mild stimulation of the nervous system's corrective reactions. The changes in balance parameters were more pronounced in the case of open eyes than in that of closed eyes. On the other hand, opposite changes were noted in the control group. After 4 weeks of therapy, the increase of ellipse major and minor axes was observed, indicating deterioration in static balance, but with a significant increase in sway velocity. Analysis of loading distribution on feet with open and closed eyes in both groups showed a small and non-significant tendency to reduce the amount of uninvolved limb loading after therapy, which may indicate gradual improvement in limb loading symmetry. However, in the control group, we have noted load transfer from the backfoot to the forefoot, which is not beneficial and is indicative of pathological lower-limb loading patterns. This was not observed in the experimental group, which may indicate that after training with the exoskeleton, load distribution within the limbs was better than after classical rehabilitation. As was reported, the appropriate load distribution should be 33% of backfoot load and 66% of forefoot load (27). In addition, as a result of training with the exoskeleton, patients significantly increased walking distance and the number of steps performed during a training unit.

The increase of forefoot loading after 4 weeks of therapy observed in the control group may indicate an increase in tension of the triceps surae and the occurrence of a spastic extensional pattern of the lower limb. This was not observed in the experimental group; therefore, we have hypothesized that it may be associated with proper muscle activation during training with the Ekso GT exoskeleton, where larger symmetry of gait pattern is preserved than when without the support of the robot. It was reported that gait with the exoskeleton induces more symmetrical activity of the lower-limb muscles, which is comparable to physiological walking and may stimulate the recovery of proper limb loading (28, 29). Therefore, the above observations may suggest that the pattern of lower-limb loading after rehabilitation with the exoskeleton is more physiological.

In the available literature, there are no studies which assess changes in balance among patients rehabilitated using an exoskeleton. Most authors have reported various aspects of gait (11, 13, 28, 30), and only a few papers present some data about changes in the balance, but only based on subjective scales as the Berg Balance Scale (13, 14). There are no studies in which changes in balance due to rehabilitation with the exoskeleton were examined using an objective tool such as a stabilometric platform.

Kubot et al. (14), in 38 patients with movement disorders (including 12 patients after stroke), evaluated balance using the Berg Balance Scale. Patients were rehabilitated using the Hybrid Assistive Limb (HAL) exoskeleton for 8 weeks, two times a week for 90 min. After therapy, the improvement in balance was noticeable, but the change was not significant. Moreover, in this study, there was no control group; thus, the effects of the intervention could not be compared (14). Similar observations were noted by Kawamoto et al. (6), who observed significant improvement in 16 patients after stroke regarding balance assessed with the Berg Balance Scale following rehabilitation with the HAL exoskeleton. The therapy included 16 sessions lasting 20–30 min, twice a week. Although the improvement of balance in the study group was significant, nonetheless, the lack of a control group made those results inconclusive. The study by Yoshimoto et al. (30) showed significant improvement in the Timed Up and Go test in chronic stroke patients and in the functional reach test and the Berg Balance Scale after training in the HAL exoskeleton (once a week for 8 weeks, 20 min per session). The control group underwent conventional physical therapy for gait disturbances, but significant differences in balance were not observed (30). Also, the research of Hornby et al. (13) or Hidler et al. (31), comparing the effects of conventional rehabilitation with treatment on the Lokomat robot, did not show any significant differences in balance assessed by the Berg Balance Scale (13, 31).

The change in balance assessed with the Berg Balance Scale (13, 14, 31), and in our own study with the stabilometric platform, did not clearly show which treatment method was more effective in patients after stroke. However, we have noted some trends showing that treatment with the exoskeleton may be more beneficial in some patients. This may indicate that to improve balance, it is necessary to stimulate the balance system itself during therapy or that the therapy duration should be longer than 4 weeks.

Patients after stroke are a very heterogeneous group in terms of functional capabilities. This condition depends on the extent of the stroke, involved areas of the brain, the time elapsed from the stroke incident, and rehabilitation conducted so far (1, 3). The main goals of treatment in patients after stroke are recovery of gripping function of the upper limbs and locomotion. Therefore, there are very few studies evaluating general functional status of patients after stroke treated with robot-assisted devices (32).

Mayr et al. (32) evaluated the impact of training with the Lokomat robot using the RMI and conventional training with a therapist on changes in the functional status of patients after stroke. They observed greater improvement in the subjects treated with the robot than in those subjected to classical therapy.

In our study, significant changes after therapy occurred in both groups; therefore, we suggest that both forms of the applied treatment lead to improvement in patients' functional status. However, in the experimental group, the improvement was observed in a larger number of categories assessed with the BI and with RMI, which may suggest the potentially greater impact of training on functional improvement with the Ekso GT exoskeleton than with classical rehabilitation. Unfortunately, the groups in our study differed significantly in some of the categories in both RMI and BI at baseline. However, patients in the experimental group were functionally weaker at baseline; therefore, it is possible that they had greater potential for improvement, and that is why their recovery of functional status was better than in the control group.

Studies have shown that the use of an exoskeleton improves gait function in patients after stroke (11, 25, 32–34). However, it cannot be unequivocally stated that the therapeutic effect is better than in the case of using classical forms of rehabilitation with a physiotherapist. In our study, significant improvement in walking distance in the exoskeleton during subsequent training sessions in patients trained with the Ekso GT exoskeleton was observed, but it should be noted that this was measured during a robot-assisted walk. Among the available literature, in some works, the results speak in favor of exoskeletons (28, 32, 34, 35), but in others, conventional rehabilitation seems to be better (13, 31). There is also a third group of studies that, like our work, do not clearly show the superiority of one method over the other (29). Clinical trials demonstrated that powered robotic exoskeletons can be used safely as gait training intervention for stroke. Preliminary findings suggest that exoskeletal gait training is equivalent to traditional therapy for chronic stroke patients, while subacute patients may experience added benefits from exoskeletal gait training (12). Also, the systematic review by Bruni et al. (35) supported the use of robot-assisted therapy in stroke patients, but when it is coupled with conventional physical therapy. They have underlined that this provides the opportunity to perform more intensive, repetitive, and task-oriented training than it would be possible with the conventional overground walking alone (35).

There are also some limitations of this study which should be addressed. Due to the nature of the intervention, blinding of the subjects was not possible. Additionally, despite the random selection of patients, the groups were heterogeneous and differed in some variables at baseline; therefore, this should be considered during interpretation of the results. It should be emphasized that patients after ischemic stroke are very heterogeneous in terms of experienced symptoms as well as their severity. More subjects should probably be included in future studies, which could minimize the problem with group heterogeneity. The main goals of treatment in patients after stroke are recovery of gripping function of the upper limbs and locomotion. But the absence of a direct measure of walking ability is also a limit of this study, and it will be investigated in further studies.

## Conclusions

Based on our results, we may conclude that both the 4 weeks of treatment with the Ekso GT exoskeleton and 4 weeks of conventional rehabilitation led to improvement in functional status of patients after ischemic stroke. However, in the experimental group, improvement was observed in a larger number of categories, which may suggest the potentially greater impact on functional status of treatment with the exoskeleton than with classical rehabilitation. Also, both forms of rehabilitation caused significant changes in balance, but we have noted some trends indicating that treatment with the exoskeleton may be more beneficial in some patients. The load transfer from the backfoot to the forefoot observed in the control group was an unfavorable phenomenon. Because this tendency was not observed in the experimental group, it may suggest the greater therapeutic effectiveness of the exoskeleton in relation to classical rehabilitation. We have suggested that the Ekso GT exoskeleton may be a promising tool in the rehabilitation of patients after stroke.

## Data Availability Statement

The datasets used and/or analyzed during the current study are available from the corresponding author on reasonable request.

## Ethics Statement

This study was approved by the Ethical Committee of Regional Medical Chamber in Krakow (77/KBL/OLI/2014). Before participation all participants received a detailed description of the study, had an opportunity to ask questions, and signed a written informed consent.

## Author Contributions

AR: study concept and design, patient recruitment, clinical management, data collection, literature search, data interpretation, and writing and editing the manuscript. AM: study concept and design, literature search, clinical management, data analyses and interpretation, statistical analyses, and writing and editing the manuscript. ŁO: study concept and design, literature search, clinical management, data interpretation, and editing the manuscript. AS and RK: study concept and design, data interpretation, and editing the manuscript.

### Conflict of Interest

The authors declare that the research was conducted in the absence of any commercial or financial relationships that could be construed as a potential conflict of interest.

## Footnotes

^1^EKSO GT User Manual, Ekso Bionics, USA.

## Abbreviations

COP: center of pressure
RMI: Rivermead Mobility Index
BI: Barthel Index.
